# A novel autophagy-related subtypes to distinguish immune phenotypes and predict immunotherapy response in head and neck squamous cell carcinoma

**DOI:** 10.17305/bb.2023.9094

**Published:** 2023-12-01

**Authors:** Bo Ma, Hui Li, Mingzhu Zheng, Rui Cao, Riyue Yu

**Affiliations:** 1 Department of Stomatology, Beijing Shijitan Hospital, Capital Medical University, Beijing, China; 2 Department of Urology, Beijing Friendship Hospital, Capital Medical University, Beijing, China

**Keywords:** Head and neck squamous cell carcinoma (HNSCC), immune phenotype, immunotherapy, tumor microenvironment (TME), tumor mutation burden (TMB)

## Abstract

Both the absence of autophagy and excessive autophagy is double-edged sword in tumorigenesis. Due to the specificity of autophagy, its role in head and neck squamous cell carcinoma (HNSCC) is still unclear. In this study, we established five autophagy-related patterns in 1165 HNSCC patients with distinct cellular and molecular characteristics. Additionally, we developed a new scoring system (ATPscore) based on the differentially expressed genes (DEGs) among these five patterns, to represent the individual autophagy regulation pattern. ATPscore was shown to be significantly correlated with tumor immune microenvironment (TIME) infiltration, immune phenotypes, molecular subtypes, and genetic variations. We further found that ATPscore was both an independent prognostic factor and a potent predictor of clinical response to immune-checkpoint inhibitors (ICIs)-based immunotherapy. We further verified the value of key gene *SRPX* in ATPscore in HNSCC cell lines with the in-depth research of ATPscore and found that it is closely related to immune subtypes, molecular subtypes, and immune activation-related markers. Our research could help us to understand the underlying mechanisms of tumor immunity and provide a solid foundation for the combination of autophagy-targeted therapies with immunotherapies for clinical application in HNSCC.

## Introduction

Head and neck squamous cell carcinoma (HNSCC) is one of the most common malignancies worldwide, with nearly 500,000 new cases and 350,000 deaths [1]. HNSCC is a heterogeneous disease that originates from the different sites of the upper aerodigestive tract, including oral cavity, oropharynx, larynx, or hypopharynx. Although combination therapy improved the prognosis of HNSCC patients, the five-year survival rate of patients with HNSCC is still less than 50% [2, 3]. Thus, the lack of rapid improvement in patient survival prompted us to further investigate the molecular landscape of HNSCC.

Autophagy is a constitutively conserved physiologic catabolic process that harvests energy and nutrients from cellular components through the degradation and recycling of damaged organelles and macromolecules [4–6]. Under normal conditions, autophagy activates to maintain cellular homeostasis by inducing nonselective bulk degradation or by selectively targeting cytoplasmic components via cargo-specific autophagy receptors [7, 8]. However, autophagy plays a dual role in tumorigenesis in a context-dependent manner [9]. A lack of autophagy could trigger the accumulation of genotoxic cellular waste and induce genetic and chromosomal alterations, subsequently facilitating the transformation of precancerous cells and the formation of mature cancer cells [10]. However, excessive autophagy continuously recycles remodeling components and replenishes energy supply, allowing cancer cells to escape damage from the immune system or targeted drugs and promote tumor progression [11, 12]. All this indicates that autophagy is a double-edged sword in tumorigenesis, which has already been verified in some preclinical cancer models [13]. In HNSCC, some reported that increased levels of cytoplasmic p62, which indicated inhibition of autophagy, were correlated with reduced overall and disease-specific survival [14]. Other groups demonstrated that patients with poor clinical outcomes had higher levels of LC3-II, suggesting reactivation of autophagy [15]. However, Zhou et al. showed that radiation-induced autophagy could enhance the survival of CNE-2 cells, which was further counteracted when autophagy was inhibitied with chloroquine, which resulted in increase in cell death [16]. We noticed a paradox in HNSCC, in which autophagy played a controversial role in tumorigenesis, but the underlying mechanism has not been reported.

Currently, we are experiencing a striking shift from combination therapy based on chemotherapy and radiation toward more precise approach based on immunotherapy with immune-checkpoints inhibitors (ICIs). Recent studies have reported that autophagy significantly controls the immune response. Autophagy can activate receptors, such as Toll-like receptors (TLRs) and nucleotide oligomerization domain (NOD)-like receptors (NLRs) to induce natural killer (NK) T cells activation, cytokine production, and phagocytosis in innate immunity. Furthermore, autophagy can also provide an abundance of antigens for MHC class II molecules, including HLA molecules toward dendritic cells for cross-priming to CD8+ T cells in adaptive immunity [17, 18]. Moreover, autophagy can facilitate, promote, or inhibit the proliferation and differentiation of a variety of immune cells or the secretion of a wide range of cytokines to modulate the tumor immune microenvironment (TIME) homeostasis. Conversely, certain cytokines and immune cells also exhibit a significant influence on autophagy function [19, 20]. Furthermore, immune checkpoints, including indoleamine 2,3 dioxygenase (IDO), cytotoxic T-lymphocyte-associated protein 4 (CTLA-4), and programmed cell death protein 1 (PD-1), have been shown to regulate tumor immune tolerance through autophagy pathways. Accumulating evidence has shown that autophagy could interact with TIME immune cells and cytokines to enhance or attenuate the immunotherapy response, providing a novel target for combination with immunotherapy [21]. However, definite correlation between autophagy and TIME has not yet been studied, and a comprehensive and systematic analysis is urgently needed.

In the present study, we integrated the transcriptional and genetic profiles of several cohorts to systematically analyze autophagy-related patterns and established an autophagy phenotype-related signature (ATPscore) for individuals. Meanwhile, we verified the value of the key gene *SRPX* in ATPscore in the HNSCC cell line. As a result, we found that distinct autophagy-related patterns were highly correlated with TIME infiltration, immune phenotypes, molecular subtypes, and clinicopathological characteristics, and that ATPscore was a robust independent prognostic and predictive factor for clinical outcome of ICIs immunotherapy.

## Materials and methods

### Data collection and processing

Publicly available transcriptional datasets for HNSCC were systematically searched. Eleven datasets, including GSE6791, GSE30784, GSE39366, GSE41613, GSE42743, GSE65858, GSE40774, GSE84846, E-MTAB-1328, E-TABM-302, and TCGA-HNSCC were included (Table S1). Finally, 6 datasets (GSE6791, GSE30784, GSE40774, GSE84846, E-TABM-302, and TCGA-HNSCC) with 1165 samples were included in our study after filtering out the samples without complete prognosis information. The above datasets were downloaded from Gene-Expression Omnibus (GEO) (https://www.ncbi.nlm.nih.gov/geo/) and ArrayExpress (www.ebi.ac.uk/arrayexpress/) database. Raw signal data were processed with the RMA algorithm background correction, log2 transformation, quantile normalization, and annotation by the “*Affy*” package in R [22]. The relative expression of each gene symbol was annotated as highest when several probes mapped to a single gene symbol. Then, the “*ComBat*” algorithm of “*sva*” package in R, which reduced the likelihood of batch effects of nonbiological technical biases from each dataset [23], was utilized to merge the five microarray datasets (GSE6791, GSE30784, GSE40774, GSE84846, and E-TABM-302) as a meta-HNSCC cohort. The level 3 fragments per kilobase per million (FPKM) data of the TCGA-HNSCC dataset were downloaded from the TCGA Genomic Data Commons (GDC) data portal (https://portal.gdc.cancer.gov/). Transcripts per kilobase million (TPM) values were transferred from the FPKM values to represent the relative expression of each gene symbol, which is more similar to gene expression from microarrays and more comparable between samples [24]. Detailed information on the clinicopathological characteristics for the TCGA-HNSCC dataset can be found in Table S2. Somatic mutation data processed with the MuTect2 algorithm for the TCGA-HNSCC cohort were downloaded from the GDC (https://portal.gdc.cancer.gov/) using the “*TCGAbiolinks*” package in R [25]. The total number of mutations counted in the whole exon territory was set at 38 Mb, according to a previous study [26]. Moreover, we also enrolled IMvigor210 (mUC) cohort, which included patients with metastatic urothelial cancer receiving programmed death-ligand 1 (PD-L1) inhibitor atezolizumab, to validate the results we found in HNSCC. Raw gene expression and clinical data from the IMvigor210 (mUC) cohort were retrieved using the “*IMvigor*” package in R (http://research-pub.gene.com/IMvigor210CoreBiologies) [27]. The detailed clinical information of the IMvigor210 (mUC) cohort can be found in Table S3. Data were analyzed with the R (version 3.5.3) and Bioconductor packages.

### Unsupervised consensus clustering for autophagy-related patterns (ATPclusters)

Autophagy-related genes (ATGs) were obtained from the Human Autophagy Database (HADb, http://www.autophagy.lu/). Then, ATGs were subjected to an unsupervised consensus clustering algorithm (K-means) based on the Euclidean distance and Ward’s linkage. This analysis was performed to identify distinct autophagy-related patterns (ATPclusters) [28]. The “*ConsensuClusterPlus*” package in R was applied to perform this procedure and repeated 1000 times to guarantee the stability of the classification [29, 30].

### Differentially expressed genes (DEGs) between ATPclusters

DEGs were screened out by comparing the patterns with different function annotations using the “*edgeR*” package in R [31], which implements an empirical Bayesian approach to evaluate the changes in gene expression in different groups. The significance criteria for determining DEGs were set as a false discovery rate (FDR) < 0.05 and |log2FC| > 1.0.

### Estimation of infiltrating immune cells

Single sample gene set enrichment analysis (ssGSEA), which evaluates the variation in pathway and biological process activity in a single sample, was utilized to estimate the relative amount of TIME immune cells in HNSCC using the “*GSVA*” package in R [32, 33]. The basic unit for ssGSEA is a gene set, consisting of genes that share common biological function, chromosomal location, or genetic regulation [34]. TIME infiltrating immune cell types, such as innate immune cells (dendritic cellss, eosinophils, mast cells, macrophages, NK cells, neutrophils, etc.) and adaptive immune cells (B cells, T cells, T helper cells, CD8+ T cells, regulatory T [Treg] cells and cytotoxic cells, etc.) were derived from the study of Charoentong et al. [35] and Bindea et al. [36]. The normalized enrichment score (NES) from the ssGSEA was regarded as the relative amount of TIME infiltrating immune cells in HNSCC.

### Function annotation and pathway enrichment analyses

A series of gene sets were curated from Mariathasan et al. to represent biological processes related to immune activation, stromal activation, DNA damage repair, and immune checkpoints, including: (a) Angiogenesis; (b) Antigen processing machinery; (c) Base excision repair; (d) CD8+ T effector; (e) Cell cycle; (f) Cell cycle regulators; (g) DNA damage repair 1 and 2; (h) DNA replication; (i) Epithelial-mesenchymal transition (EMT) 1, 2, and 3; (j) Fanconi anemia; (k) Homologous recombination; (l) Immune checkpoint; (m) Mismatch repair; (n) Nucleotide excision repair; (o) Pan-fibroblast TGF-β response signature (Pan-F-TBRS), and (p) WNT targets [27, 37, 38].

### Construction of autophagy phenotype-related signature (ATPscore)

In order to define autophagy phenotype in individual patients, a set of scoring system was required. The DEGs between patterns were subjected to univariate Cox analysis to generate candidate prognostic DEGs at cut-off *P* value < 0.01. For dimension reduction, the least absolute shrinkage and selection operator (LASSO)-Cox regression algorithm for prognostic DEGs was used to construct an optimal autophagy-phenotype-related signature (ATPscore) using the “*glmnet*” package in R [39]. The optimal values of the penalty parameter *λ* were determined by 10 cross-validations. The ATPscore of each sample was defined by the relative expression of candidate prognostic DEGs within the model and their Cox coefficients. ATPscore ═ ∑_(*i* ═ 1)_^*n*^ (coef*i* × Expr*i*), where Expr*i* is the relative expression of DEG in the signature for patient *i* and coef*i* is the LASSO-Cox coefficient of DEG *i*.

### Cell culture and silencing of *SRPX* in head and neck squamous cell carcinoma (HNSCC) cells

The HNSCC cell lines FaDu and CAL 27 were used for the experiment. The FaDu cells were maintained in minimal essential medium (MEM; Gibco, China) and CAL 27 cells were maintained in Dulbecco’s Minimal Essential Medium (DMEM; Gibco, China) supplemented with 1% penicillin G sodium/streptomycin sulfate and 10% fetal bovine serum (FBS; Gibco, Australia). All cells were grown in a humidified atmosphere consisting of 5% CO_2_ and 95% air at 37 ^∘^C [40]. *SRPX*-target-specific-small interfering RNA (siRNA) and negative-control-siRNA were all synthesized by Genepharma Ltd. in Suzhou, China. FaDu and CAL 27 were transfected either by *SRPX*-target-specific-siRNA (*SRPX* KD) or negative-control-siRNA (NC) using Lipofectamine 2000 (Thermofisher, USA) according to the manufacturer’s protocol. The sequences of each siRNA were as follows: siRNA-1:5’-GCCATGCCAGCAAATGGAGGGTTTA-3’, siRNA-2:5’-AGAGACACAGCAGATGGAATTCTTA-3’, siRNA-3:5’-CACAGCAGATGGAATTCTTACTGAT-3’, si-NC:5’-UUCUCCGAACGUGUCAGGUTT-3’. After transfection for 72 h, alterations of *SRPX* at the transcriptional levels were validated by quantitative real-time PCR (qRT-PCR).

### Total RNA isolation and reverse transcription and quantitative real-time PCR (qRT-PCR)

Total RNA from the HNSCC cells was extracted using the Qiagen RNeasy Mini Kit (Cat. #74101, Qiagen, Germany) combined with the QIAshredder from Qiagen (Cat. #79654, Qiagen, Germany) according to the manufacturer’s protocol. Then, DNase I was used to remove the contamination of genomic DNA in each RNA sample (RNase-Free DNase Set, Cat. #79254, Qiagen, Germany). First-strand cDNA and real-time polymerase chain reaction (PCR) were performed using the ReverTra Ace qPCR RT Kit (Toyobo, China) and iQTM SYBR^®^ Green Supermix (Bio-Rad, China), respectively. The optimal annealing temperatures and PCR conditions for each primer were optimized with gradient PCRs using an iCycler (Cat. #CFX Connect, Bio-Rad, USA). The *GAPDH* alleles were used as an internal reference. *SRPX* primers: 5’-ATCAAGGTGAAGTATGGGGATGT-3’ (forward), 5’-GTTTGACTGGCAGATCAGTAGG-3’(reverse). *GAPDH* primers: 5’-ACAACTTTGGTATCGTGGAAGG-3’ (forward), 5’-GCCATCACGCCACAGTTTC-3’(reverse).

### Detection of proliferation and migration of HNSCC cells

Distinct HNSCC cells were seeded in 96-well plates at 5000 cells per 200 µL of medium. Next, HNSCC cells were incubated in the medium for another 4 days until the 20 µL of Cell Counting Kit 8 (CCK-8) (10 mg/mL) was added. The absorbance was measured at 450 nm using a microplate reader (Cat. #SpectraMax M2, Molecular Devices, USA) after the cells were incubated at 37 ^∘^C for 1 h. When HNSCC cells grew to 95% confluence, a wound was created in the cell monolayer using a 200 µL pipette tip. Then, 0.5% FBS medium was used to allow cells to migrate into the gap without the influence of serum. Four different equidistant points of the scratched area were photographically measured and imaged by an inverted phase contrast microscope (Leica, Cat. #DMI1) at 0, 24, and 48 h. A 24-well transwell chamber system (Corning, USA) with 8.0 µm pore size was used to perform the migration assay. Distinct HNSCC cells were suspended in a serum-free medium at a density of 50,000 cells per 100 µL and seeded in the upper chamber insert, while the lower chamber was filled with 10% FBS medium. After incubation for 24 h at 37 ^∘^C, the cells in the upper insert were removed, and the cells that had migrated to the lower side were fixed with 4% PFA and stained with crystal violet. Then, the migrated cells were observed and counted using the inverted phase contrast microscope.

### Ethical statement

The entire research followed the principles outlined in the Declaration of Helsinki.

### Statistical analysis

Data were expressed as means ± SD. All analyses were performed at least three times and represent data from three individual experiments. Two-tailed student’s *t*-tests were used to assess the statistical significance of differences between the groups. Statistical significance of variables between two groups or more than two groups was analyzed by Wilcoxon tests or Kruskal–Wallis tests, respectively. Differences between survival curves for each group were determined by Kaplan–Meier analysis with log-rank test using the “*survminer*” package in R. The distance between different parameters was computed by Pearson and distance correlation analyses. Independent prognostic factors were identified by univariate and multivariate Cox proportional hazard analysis and visualized with the “*forestplot*” package in R. ATPscore was integrated with other independent factors to establish the nomogram and calibration curves using the packages “*rms*”, “*nomogramEx*”, and “*regplot*” in R. According to Iasonos’ suggestion, decision curve analysis (DCA) was used to evaluate the clinical utility of the nomogram [41]. The mutation landscape of patients in the TCGA-HNSCC cohort was visualized with a waterfall plot using the packages “*maftools*” [42] and “*complexheatmap*” [43] in R. Contingency tables, such as the ICIs targeting immunotherapy response, were analyzed by two-sided Fisher’s exact tests. All statistical analyses were performed with R software 3.5.3. Statistical significance was set at *P* < 0.05.

## Results

### Characterization of autophagy-related patterns (ATPclusters) in HNSCC

**Figure 1.**
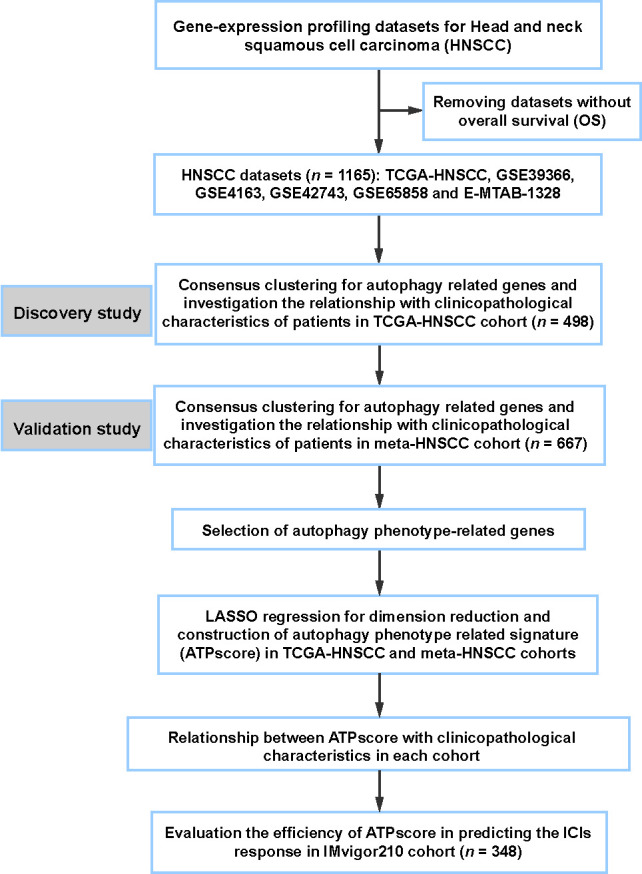
**Overview of workflow and study design**. LASSO: Least absolute shrinkage and selection operator; ICI: Immune-checkpoint inhibitor.

**Figure 2.**
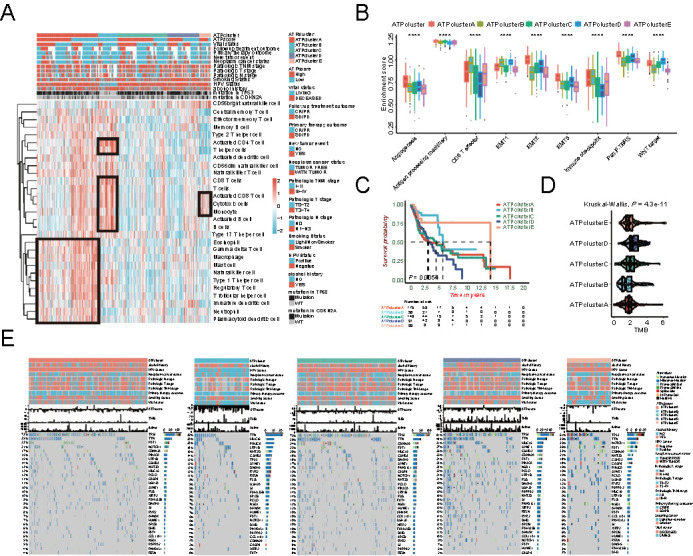
**Clinicopathological characteristics in distinct autophagy-related patterns (ATPclusters).** (A) Hierarchical clustering of TIME landscape in the TCGA-HNSCC cohort. Rows represent relative amount of each immune cell, and columns represent HNSCC samples. Red represents relatively upregulated and blue represents relatively downregulated immune cells; (B) Difference in the enrichment of specific signatures to represent biological processes related with stromal-activation and immune-activation among five distinct autophagy-related patterns in the TCGA-HNSCC cohort; (C) Kaplan–Meier survival curves for distinct autophagy-related patterns in the TCGA-HNSCC cohort; (D) Differences in TMB among different autophagy-related patterns in the TCGA-HNSCC cohort. The upper and lower ends of the boxes represent interquartile range of values. The lines in the boxes represent median value; (E) Distribution of top 30 variant mutated genes among five distinct autophagy-related patterns in the TCGA-HNSCC cohort. The genetic alterations types include frame shift del, frame shift ins, in frame del, in frame ins, missense mutation, multi-hit, nonsense mutation, and splice site. The upper bar plots indicate ATPscore, TMB, and OS time. The number on the left and right bar plots show the mutation frequency of each gene. TIME: Tumor immune microenvironment; HNSCC: Head and neck squamous cell carcinoma; TMB: Tumor mutation burden; OS: Overall survival.

The workflow of this study is shown in Figure 1. The TCGA-HNSCC cohort was used as the training set to identify the autophagy-related patterns (ATPcluster) using the “*ConsensuClusterPlus*” package in R. We found that ATGs could successfully from five ATPclusters with high stability in the TCGA-HNSCC cohort, including 175 cases in pattern A, 56 cases in pattern B, 143 cases in pattern C, 191 cases in pattern D, and 33 cases in pattern E, which was termed as ATPcluster A–E, respectively (Figure S2A). Unsupervised hierarchical clustering demonstrated that ATGs were significantly differentially expressed among ATPcluster A–E in the TCGA-HNSCC cohort (Figure S2B). Moreover, Kaplan–Meier survival curves showed that ATPcluster A–E displayed a completely different survival benefits in which patients with ATPclusters B and E had a better prognosis than other clusters (Log-rank test, *P* ═ 0.0054; Figure 2C).

### Tumor immune microenvironment (TIME) landscape in distinct ATPclusters

As autophagy played a dual role in TIME, the landscape of TIME was calculated via the ssGSEA algorithm and shown in the cluster heat map (Table S4). The relative amount of TIME immune cells was strikingly different in distinct ATPclusters as follows (Figure 2A): ATPcluster A exhibited high infiltration with almost all immune cells; ATPcluster B was remarkably rich in effector immune cells, but less infiltrated with immunosuppressive cells; ATPclusters C and D displayed low infiltration with all immune cells; ATPcluster E was characterized by high infiltration with activated CD8+ and cytotoxic cells but low infiltration with regulatory T cells, macrophages, and mast cells.

### Distinct ATPclusters exhibited different immune phenotypes

We found that ATPcluster A was highly infiltrated with immune cells, but patients with this pattern had a worse prognosis. Recent study has determined three immune phenotypes of tumors: desert, excluded, and inflamed. Immune inflamed phenotype was characterized as high infiltration with immune cells, while immune desert phenotype showed the opposite situation. Immune excluded phenotype was considered cytotoxic T cell suppressive by featuring the infiltration with abundant immune cells, which were located in the stroma surrounding the core tumor niche rather than penetrating its parenchyma [44]. We then included a specific gene set from Mariathasan et al. to investigate the enrichment of key signaling pathways associated with immune phenotypes. From the TIME landscape and function annotation, ATPcluster A was recognized as an immune-excluded phenotype as stromal-related signaling pathways, including angiogenesis, epithelial-mesenchymal transition (EMT), WNT target, and pan-fibroblast TGF-β response signaling pathways (pan-F-TBRS) were strikingly activated, which could hamper the beneficial effect of high immune cell infiltration (Figure 2B and Table S5). Furthermore, ATPclusters B and E were remarkably associated with the induction of immune activation signaling pathways, including antigen processing machinery, CD8+ T effector, and immune checkpoint. However, they were deactivated in stromal-related signaling pathways, representing the characteristics of an immune-inflamed phenotype (Figure 2B and Table S5). Moreover, ATPclusters C and D were more likely to have an immune-desert phenotype (Figure 2B and Table S5).

### Tumor somatic mutations in distinct ATPclusters

The relationship between somatic mutations and autophagy was also measured. We found that ATPcluster D had the highest tumor mutation burden (TMB), while ATPcluster B was associated with the lowest TMB (Figure 2D). Moreover, top 30 highly variant mutant genes were utilized to plot the somatic mutation landscape among distinct ATPclusters in patients with HNSCC. ATPcluster D displayed the highest mutation rate of top 30 mutant genes, especially for *TP53* (Figure 2E), which was identified as key gene in tumorigenesis of HNSCC [45]. But ATPclusterB only showed small amount of *TP53* mutations, which was consistent with the TMB calculation. All the above results demonstrated that autophagy-related patterns correlated both with TIME infiltration and tumor mutation landscape, which underlies the indispensable role of autophagy in the HNSCC development.

### Validation of ATPclusters in meta-HNSCC cohort

**Figure 3.**
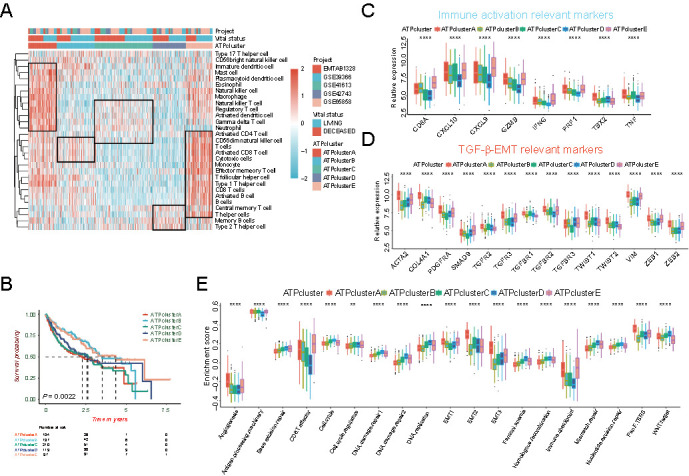
**Characterization of autophagy-related patterns (ATPclusters) in the meta-HNSCC cohort.** (A) Hierarchical clustering of TIME landscape in the meta-HNSCC cohort. Rows represent relative amount of each immune cell, and columns represent HNSCC samples. Red represents relatively upregulated and blue represents relatively downregulated immune cells; (B) Kaplan–Meier survival curves show the difference in prognosis advantage among five distinct autophagy-related patterns in the meta-HNSCC cohort; (C and D) Difference in the expression of immune activation relevant markers (C), TGF-β/EMT relevant markers (D) among five distinct autophagy-related patterns in the meta-HNSCC cohort; (E) Difference in the enrichment of immune activation, stromal activation, and DNA damage repair (DDR) relevant signatures among five distinct autophagy-related patterns in meta-HNSCC cohort. The upper and lower ends of the boxes represent interquartile range of values. The lines in the boxes represent the median value and the black dots show outliers. The asterisks represent the statistically significant *P*-value (*P < 0.05; **P < 0.01; ***P < 0.001; ****P < 0.0001). HNSCC: Head and neck squamous cell carcinoma; TIME: Tumor immune microenvironment; EMT: Epithelial-mesenchymal transition; TGF-β: Tumor growth factor beta.

Unsupervised consensus clustering also identified five ATPclusters with differential transcriptional profile of ATGs in meta-HNSCC cohort (Figure S2A and S2B). Moreover, Kaplan–Meier survival curves demonstrated that the prognosis of five distinct ATPclusters were strikingly different, with patients with ATPclusters B and E living longer and patients with ATPclusters A, C, and D were associated with worse survival (Log-rank test, *P* ═ 0.0022; Figure 3B).

Furthermore, a similar TIME landscape among five distinct ATPclusters of the TCGA-HNSCC cohort was determined in meta-HNSCC cohort as follows (Figure 3A and Table S6): ATPclusters A and B were more infiltrated with immune cells, while ATPclusters C and D were less infiltrated. Compared with effector immune cells (CD4+, CD8+ T cells, and cytotoxic cells), ATPcluster A exhibited an abundance of immunosuppressive cells (Treg cells, macrophages, and mast cells). The opposite situation was found in ATPclusters E and B, where the ratio of cytotoxic T lymphocytes (CTL), including activated CD8+ T cells and cytotoxic cells to Treg cells was higher among five ATPclusters. ATPclusters C and D displayed the lowest infiltration of effector immune cells among the five ATPclusters.

Moreover, the ssGSEA of specific gene sets also showed a similar trend as the TCGA-HNSCC cohort. ATPcluster A was enriched in both immune and stromal activation signaling pathways, which could be recognized as immune-excluded phenotype. ATPclusters B and E were more prominently enhanced in immune activation gene sets, such as CD8+ T effector and immune checkpoint, which was more likely to immune-inflamed phenotype. And ATPclusters C and D were slightly associated with DNA damage response (DDR)-related signaling pathways but were less enriched in immune infiltration signaling pathways, which was the characteristic of immune-desert phenotype (Figure 3E and Table S7).

Then, the major markers representing the immune phenotypes-related signaling pathways were subsequently curated as follows: immune activation: CD8A, CXCL10, CXCL9, GZMA, GZMB, IFNG, PRF1, TBX2, and TNF; TGF-β/EMT signaling pathway: ACTA2, COL4A1, PDGFRA, SMAD9, TGFB1/2/3, TGFBR1/2/3, TWIST1/2, VIM, and ZEB1/2. Surprisingly, the expression of the above markers showed a similar distribution as the biological processes and pathways enrichment among distinct ATPclusters (Figure 3C and 3D).

### Establishment of autophagy phenotype-related signature (ATPscore)

The above findings revealed that there were distinct autophagy-related patterns in HNSCC and demonstrated an essential role of autophagy in shaping TIME landscapes, but all analyses were performed in cohorts with patient population. Due to the heterogeneity of individuals, there was an urgent need to construct a set of scoring system to quantify the autophagy-related patterns in individual patients with HNSCC. By comparing the transcriptomic profiles of the main autophagy-related patterns, we obtained 5734 phenotype-related meta-DEGs (Table S8). Then, 383 of the 5734 meta-DEGs which were significantly correlated with prognosis were identified as autophagy phenotype candidate genes (Table S9). Furthermore, LASSO-Cox regression analysis was utilized for dimension reduction on these genes to construct an autophagy phenotype-related signature (ATPscore) which was representative of autophagy-related pattern in individuals. Finally, 11 genes were selected to create ATPscore and the formula was as follows: ATPscore ═ ACTL10*(−0.0729) + C19orf57*(−0.0292) + CHAD*(−0.0801)+ FCN2*(−0.3865) + FGB*(0.1961) + GPR174*(−0.0437) + HSF5*(−0.0126) + SERPINA5*(0.1589) + SRPX*(0.0322) + ZNF541*(−0.033) + ZNF831*(−0.3632).

**Figure 4.**
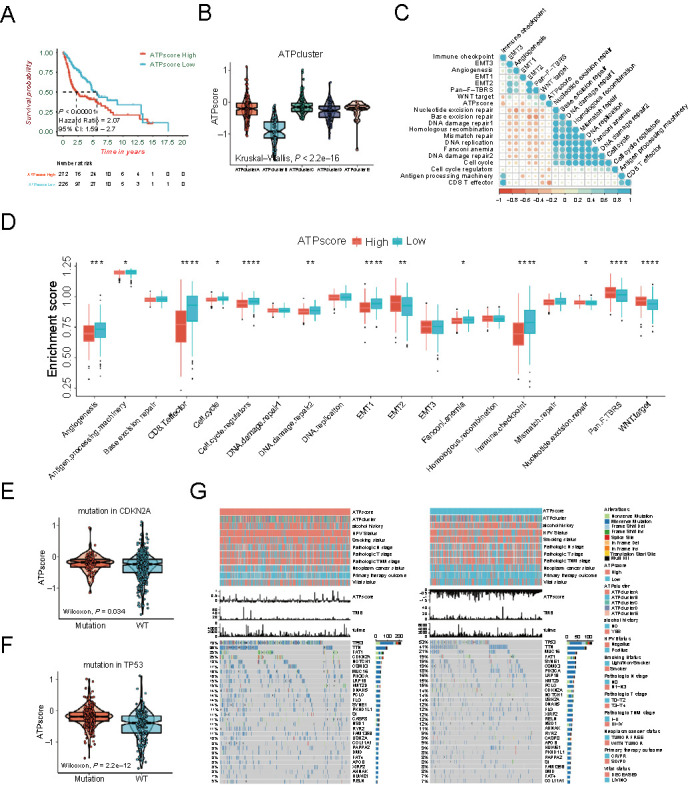
**Establishment of autophagy phenotype-related signature (ATPscore) in the TCGA-HNSCC cohort.** (A) Kaplan–Meier survival curves show the difference in prognosis between the high and low ATPscore groups in the TCGA-HNSCC cohort; (B) Difference in ATPscore among five distinct autophagy-related patterns in the TCGA-HNSCC cohort. Kruskal–Wallis test was used to compare the statistical difference between each pattern; (C) Correlation between ATPscore and stromal-activation, immune-activation, and DDR-relevant signatures in the TCGA-HNSCC cohort. Negative correlation is marked with red and positive correlation is marked with blue; (D) Difference in the enrichment of specific signatures to represent biological processes related with stromal-activation, immune-activation, and DDR between the high and low ATPscore groups in the TCGA-HNSCC cohort; (E) Differences in ATPscore between patients with *CDKN2A* mutation and *CDKN2A* WT in the TCGA-HNSCC cohort. The statistical difference was tested by Wilcoxon test; (F) Differences in ATPscore between patients with *TP53* mutation and *TP53* WT in the TCGA-HNSCC cohort. The statistical difference was tested by the Wilcoxon test; (G) The waterfall plot shows the distribution of top 30 highly variant mutated genes between the high and low ATPscore groups. The genetic alterations types are indicated in the waterfall plot annotation. The asterisks represent the statistically significant *P*-value (*P < 0.05; **P < 0.01; ***P < 0.001; ****P < 0.0001). HNSCC: Head and neck squamous cell carcinoma; DDR: DNA damage repair; WT: Wild type.

The Kaplan–Meier survival curves showed that patients with the low ATPscore lived longer than patients with the high ATPscore in the TCGA-HNSCC cohort (Log-rank test, *P* < 0.00001; Figure 4A). Then, we found that ATPscore was differentially distributed among the five ATPclusters: ATPcluster A with poor prognosis showed the highest median score while ATPcluster B with good prognosis showed the lowest median score (Kruskal–Wallis test, *P* < 2.2e-16; Figure 4B). Moreover, function annotation demonstrated that immune activation gene sets, such as antigen processing machinery, CD8+ T effector, and immune checkpoint, were strikingly induced in the low ATPscore group, while stromal-relevant signaling pathways were enhanced in the high ATPscore group (Figure 4D). Then, ATPscore was found to be negatively correlated with immune activation signature and positively correlated with the stromal activation signature through Pearson correlation analysis (Figure 4C and Table S10).

**Figure 5.**
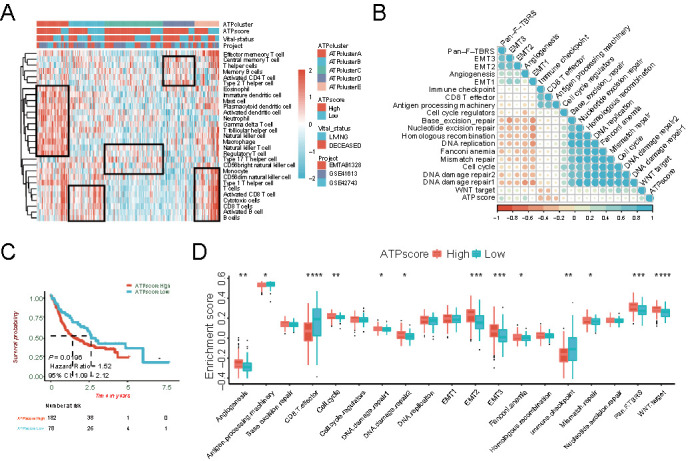
**Validation of autophagy phenotype-related signature (ATPscore) in the microarray-HNSCC cohort.** (A) Hierarchical clustering of TIME landscape in the microarray-HNSCC cohort; (B) Correlation matrix of ATPscore and stromal-activation, immune-activation, and DDR relevant signatures in the microarray-HNSCC cohort; (C) Kaplan–Meier survival curves show the difference in prognosis between the high and low ATPscore groups in the microarray-HNSCC cohort; (D) Difference in the enrichment of specific signatures to represent biological processes related with stromal-activation, immune-activation, and DDR between the high and low ATPscore groups in the microarray-HNSCC cohort. The asterisks represent the statistically significant *P*-value (*P < 0.05; **P < 0.01; ***P < 0.001; ****P < 0.0001). HNSCC: Head and neck squamous cell carcinoma; TIME: Tumor immune microenvironment; DDR: DNA damage repair; ATPscore: Autophagy phenotype-related signature.

The role of ATPscore was validated in the EMTAB1328, GSE41613, and GSE42743 meta-cohorts. Cluster heat map demonstrated that TIME infiltration distribution among five ATPclusters was similar to the meta-HNSCC cohort (Figure 5A and Table S11). Moreover, we could clearly see that the amount of effector immune cells, including CD8+ T cells and cytotoxic cells, was robustly accumulated in the low ATPscore group when compared with the high ATPscore group in ATPclusters A, B, and E (Figure 5A). The Kaplan–Meier survival curves demonstrated that patients with the low ATPscore had better prognosis than patients with the high ATPscore (Log-rank test, *P* ═ 0.0196; Figure 5C). Moreover, ATPscore was significantly positively correlated with angiogenesis, EMT, Pan-F-TBRS, and WNT targets signatures, and negatively correlated with antigen processing machinery, CD8+ T effector, and immune-checkpoint signatures (Figure 5B–5D and Table S12 and S13). All the above results strongly suggested that ATPscore is a good representative of autophagy-related patterns and is competent at distinguishing immune phenotypes in HNSCC.

### ATPscore could be utilized as an independent prognostic factor in HNSCC

Next, we wanted to clarify the association between ATPscore and clinical characteristics in HNSCC. The results showed that patients in the high ATPscore group were more likely to be a more advanced pathological TNM stage, HPV-negative, and smokers. They tended to have a treatment outcome of stable disease (SD), progressive disease (PD), with tumor, and deceased status. However, no correlation was observed with a history of alcohol consumption. The opposite patterns could be seen in the low ATPscore group (Figure S4A–S4L).

Because gene mutation and TMB were remarkably different in five ATPclusters, waterfall plots also revealed that *TP53* and *CDKN2A* were differentially mutated between the high and low ATPscore groups (Figure 4G). Kaplan–Meier survival curves demonstrated that the prognosis of patients with *TP53* mutation was robustly worse than that of patients with *TP53* wild type (WT) (data not shown). We were amazed to find that patients with *TP53* mutations displayed higher ATPscore (Figure 4F). Although mutation of *CDKN2A* was not correlated with patient survival, patients with the high ATPscore were also more likely to have *CDKN2A* mutation (Figure 4E).

**Figure 6.**
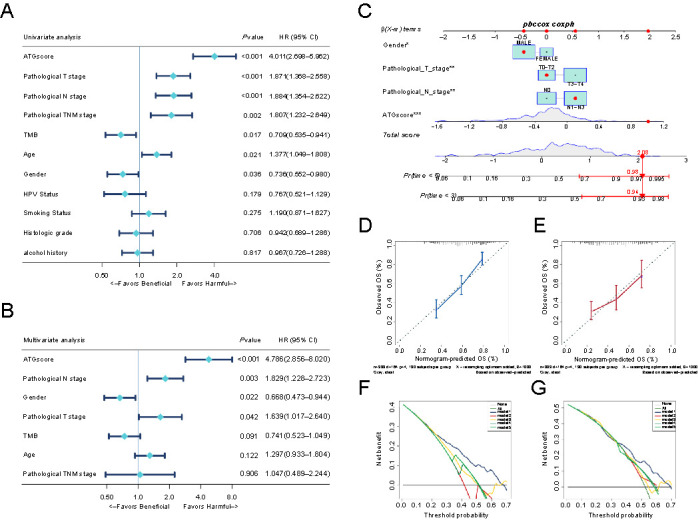
**Autophagy phenotype-related signature (ATPscore) was an independent prognosis factor in HNSCC.** (A and B) Forest plot summary of the univariate and multivariable cox regression analyses measuring ATPscore and clinicopathological characteristics. The *P* value, HR, and 95% CI are indicated in the figure; (C) Nomograms which integrated with independent prognosis factor for predicting the probability of patient mortality at 3- or 5-years OS. Blue circle is a point each parameter scored; (D and E) DCA curves for four independent prognostic factors or combination of them in OS prediction at 3-years (D) and 5-years (E); (F and G) Calibration curves of the nomogram for predicting the probability of OS at 3- (F) and 5-years (G). HNSCC: Head and neck squamous cell carcinoma; DCA: Decision curve analysis; TMB: Tumor mutation burden.

Furthermore, univariate and multivariate Cox regression analyses demonstrated that the ATPscore, pathological T stage, pathological N stage, and sex were independent factors that could be used to predict the prognosis of HNSCC patients (Figure 6A and 6B and Table S14). We then constructed a nomogram by integrating the above independent prognostic factors to serve as a clinically relevant quantitative method for clinicians to predict mortality in patients with HNSCC (Figure 6C). Using the nomogram, each patient would receive a total point by adding the points for each prognostic parameter. The patients having higher total points corresponded to a worse clinical outcome. The calibration plots and DCA demonstrated that our nomogram had a similar performance to that of an ideal model and had high potential clinical utility (Figure 6D–6G). All of these demonstrated that ATPscore could not only estimate the autophagy-related pattern of individual and characterize immune phenotypes but also further act as an independent prognostic factor in HNSCC.

### ATPscore was potent to predict clinical response to immune-checkpoint inhibitors (ICIs) immunotherapy

**Figure 7.**
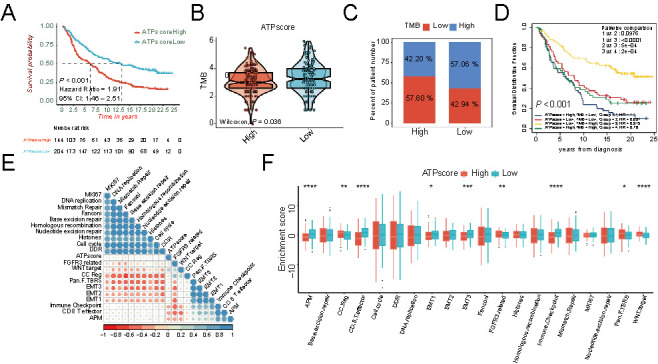
**Autophagy phenotype-related signature (ATPscore) was also efficiency in IMvigor210 dataset.** (A) Kaplan–Meier survival curves show the difference in prognosis between the high and low ATPscore groups in the IMvigor210 (mUC) cohort; (B) Difference in TMB between the high and low ATPscore groups in the IMvigor210 (mUC) cohort; (C) The proportion of TMB between the high and low ATPscore groups in the IMvigor210 (mUC) cohort; (D) Kaplan–Meier survival curves show the difference in prognosis advantage among four groups stratified by ATPscore and TMB in the IMvigor210 (mUC) cohort; (E) Correlation between ATPscore and specific signature in the IMvigor210 (mUC) cohort. Blue indicates positive correlation and red indicates negative correlation; (F) Difference in the enrichment of specific signature between the high and low ATPscore groups in the IMvigor210 (mUC) cohort. CR: Complete response; PR: Partial response; SD: Stable disease; PD: Progressive disease. CR/PR was identified as responder and SD/PD was identified as non-responder; ICI: Immune-checkpoint inhibitor; TMB: Tumor mutation burden; PD-L1: Programmed death-ligand 1.

Recently, ICIs immunotherapy has emerged as a major breakthrough in the treatment of solid tumors. The above findings demonstrated that the ATPscore could not only distinguish the prognosis of patients but also determine TIME infiltration and immune phenotype, which indirectly confirmed its potential role in predicting clinical response to ICIs treatment. Then, the IMvigor210 (mUC) cohort was used to study the role of ATPscore in evaluating immunotherapeutic benefits, which consisted of patients with metastatic urothelial cancer receiving PD-L1 inhibitor with atezolizumab. Kaplan–Meier survival curves showed that patients with the low ATPscore had a significantly better clinical outcome compared with patients with the high ATPscore (Log-rank test, *P* < 0.001, Figure 7A).

**Figure 8.**
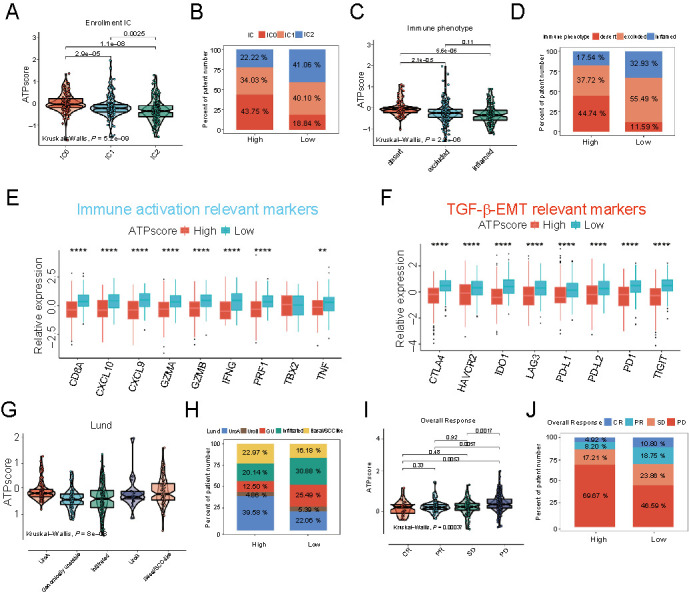
**Autophagy phenotype-related signature (ATPscore) was an efficiency tool to predict clinical response to ICIs immunotherapy.** (A) Differences in ATPscore between different PD-L1 expression on immune cells (IC) in the IMvigor210 (mUC) cohort; (B) The proportion of PD-L1 expression on immune cells (IC) subtypes between the high and low ATPscore groups in the IMvigor210 (mUC) cohort; (C) Differences in ATPscore among different immune phenotypes in the IMvigor210 (mUC) cohort; (D) The proportion of immune phenotypes between the high and low ATPscore groups in the IMvigor210 (mUC) cohort; (E and F) Immune activation relevant markers (E) and TGF-β/EMT relevant markers (F) were differentially expressed between the high and low ATPscore groups in the IMvigor210 (mUC) cohort; (G) Differences in ATPscore among different molecular subtypes in Lund molecular classification system in the IMvigor210 (mUC) cohort; (H) The proportion of Lund molecular subtypes between the high and low ATPscore groups in the IMvigor210 (mUC) cohort; (I) Differences in ATPscore between different ICIs immunotherapy clinical response groups; (J) The proportion of patients who responded to ICIs immunotherapy between the low or high ATPscore groups in the IMvigor210 (mUC) cohort. The asterisks represent the statistically significant *P*-value (*P < 0.05; **P < 0.01; ***P < 0.001; ****P < 0.0001). CR: Complete response; PR: Partial response; SD: Stable disease; PD: Progressive disease. CR/PR was identified as responder and SD/PD was identified as non-responder; ICI: Immune-checkpoint inhibitor; TMB: Tumor mutation burden; PD-L1: Programmed death-ligand 1.

A previous study reported that ICIs treatment responders were more likely to be patients with the genomically unstable (GU) subtype in the Lund classification system and TCGA II subtype in the TCGA classification system, using the IMvigor210 (mUC) cohort. Here in the cluster heat map, we found that the low ATPscore group exhibited an abundance of activated B, activated CD4+, CD8+ T cells, and cytotoxic cells, but was less infiltrated with Treg, macrophages, and mast cells in patients with GU and TCGA II subtypes (Figure S5A). Moreover, ssGSEA of specific gene sets showed that antigen processing machinery (APM), CD8+ T effector, and immune checkpoints signature, representing the immune activation, were strikingly enriched in the low ATPscore group (Figure 7F). The markers associated with immune activation showed a similar trend to function annotation (Figure 8E). However, we found that the EMT signature was slightly activated in the low ATPscore group, but Pan-F-TBRS and WNT-target signatures were strikingly enriched in the high ATPscore group (Figure 7F and Figure 8F). Most markers related to EMT/TGF-β signaling pathways were not differentially expressed between two groups, which indicated that ATPscore might not distinguish the stromal pattern in the IMvigor210 (mUC) cohort. The results of the correlation matrix were almost identical to the above findings (Figure 7E). Moreover, we were amazed to find that the patients with the immune-inflamed phenotype had the lowest ATPscore, while patients with the immune-desert phenotype had the highest ATPscore (Kruskal–Wallis test, *P* ═ 2.2e-6; Figure 7D). The number of patients with immune-inflamed phenotype was twice as high than in the low ATPscore group when compared with that in the high ATPscore group (Fisher’s exact test, *P* < 0.00001; Figure 8D). All this definitely confirmed our hypothesis that autophagy was able to shape the TIME infiltration and distinguish the immune phenotype.

Although ATPscore showed no correlation with TMB in the TCGA-HNSCC cohort, we were surprised to find that there was a negative correlation between TMB and ATPscore in the IMvigor210 (mUC) cohort (Wilcoxon test, *P* ═ 0.036; Figure 7B and Fisher’s exact tests, *P* ═ 0.033886; Figure 7C). Kaplan–Meier survival curves revealed that the survival benefit of patients with high TMB was superior to that of patients with low TMB (Log-rank test, *P* < 0.001; Figure 7D). Moreover, we combined the information of ATPscore and TMB to find that patients with low ATPscore as well as with high TMB exhibited a tremendous survival advantage over all other subgroups (Log-rank test, *P* < 0.001; Figure 7D). We then comprehensively explored the role of ATPscore in the entire IMvigor210 (mUC) cohort, as it contained much information associated with immunotherapy response. We found that the GU subtype and TCGA II subtype, which displayed high somatic mutation and more likely responded to ICIs treatment, demonstrated the lowest ATPscore when compared with other molecular subtypes in the Lund and TCGA classification systems, respectively (Kruskal–Wallis test, *P* ═ 8e-8; Figure 8G; Kruskal–Wallis test, *P* ═ 2.6e-5; Figure S5D). The number of patients with the GU subtype and TCGA II subtype in the low ATPscore group was more than twice that in the high ATPscore group (Fisher’s exact test, *P* ═ 0.010413; Figure 8H; Fisher’s exact test, *P* ═ 0.116173; Figure S5E). Furthermore, the correlation between ATPscore and the immune checkpoint PD-L1 located on tumor cells (TC) or immune cells (IC) was also measured. Surprisingly, we found that most of patients with IC2, which was correlated with better clinical outcome of immunotherapy, were concentrated in the low ATPscore group, while IC0 strikingly accumulated in the high ATPscore group (Fisher’s exact test, *P* ═ 0.000136; Figure 8A). Moreover, patients with IC2 showed the lowest ATPscore and patients with IC0 showed the highest ATPscore (Kruskal–Wallis test, *P* ═ 5.2e-9; Figure 8B). We found no difference in ATPscore among patients with TC0-TC2 (Kruskal–Wallis test, *P* ═ 0.34; Figure S5B; Fisher’s exact test, *P* ═ 0.728113; Figure S5C). In addition, we were delighted to find that patients with the low ATPscore were more likely to be ICIs treatment responders (complete remission [CR]/partial remission [PR]), while patients with high ATPscore tended to be non-responders (SD/PD) (Kruskal–Wallis test, *P* ═ 0.00037; Figure 8I). Moreover, the number of patients who responded to ICIs immunotherapy (CR/PR) was more than twice as high in the low ATPscore group as that in the high ATPscore group (Fisher’s exact test, *P* ═ 0.006009; Figure 8J). All our findings implied that there are distinct autophagy-related patterns in tumors, which could shape the TIME infiltration and immune phenotypes, as well as predict the clinical outcome of ICIs immunotherapy.

### *SRPX* was negatively correlated with HNSCC cell proliferation and migration

**Figure 9.**
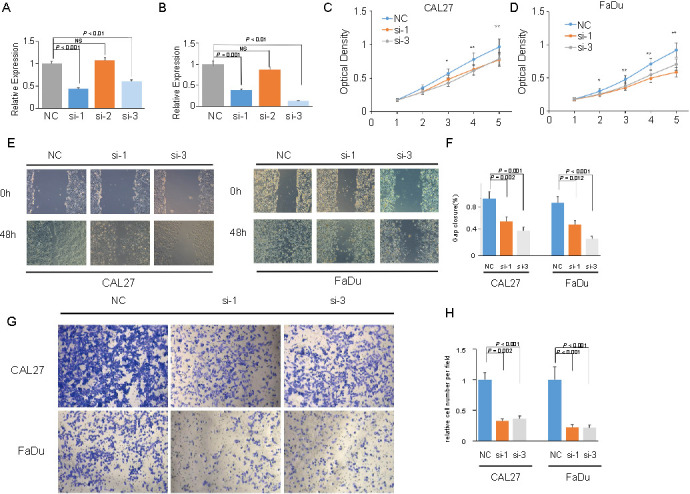
***SRPX* was negatively correlated with HNSCC cell proliferation and migration.** (A and B) Expression of *SRPX* in CAL27 (A) and FaDu (B) cell lines after siRNA transfection validated by qRT-PCR; (C and D) CCK-8 assay indicated cell proliferation of CAL27 (C) and FaDu (D) cell lines after siRNA transfection; (E and F) Wound healing assay of CAL27 (E) and FaDu (F) cell lines after siRNA transfection; (G and H) Transwell assay of CAL27 (G) and FaDu (H) cell lines after siRNA transfection. HNSCC: Head and neck squamous cell carcinoma; siRNA: Specific-small interfering RNA; qRT-PCR: Quantitative real-time PCR; CCK-8: Cell Counting Kit 8.

In order to validate our findings, we silenced the expression of *SRPX* in two HNSCC cell lines (CAL27 and FaDu). The qRT-PCR (Figure 9A and 9B) validated that si-1 and si-3 efficiently silenced the gene and protein expression of *SRPX* in HNSCC cells and was selected for subsequent study. Next, we found that *SRPX*-target-specific-siRNA (*SRPX* KD)-treated HNSCC cells grew more slowly than the control-siRNA (NC)-treated HNSCC cells, which were detected using the CCK-8 assay (Figure 9C and 9D). Additionally, wound healing (Figure 9E and 9F) and transwell assays (Figure 9G and 9H) revealed that HNSCC cells migrated significantly less after *SRPX* silencing.

## Discussion

In recent years, the key topics on cancer research have shifted from focusing on the tumor itself to studying the interaction between the tumor and its surrounding environment, commonly referred as to tumor microenvironment. Tumor microenvironment. in which tumor cells originate and live, includes not only the tumor cells themselves but also their surrounding stroma, microvasculature, and a variety of other cells, including immune cells, fibroblasts, and more, as well as the biological molecules they secrete, such as cytokines and chemokines [46]. They cooperate with each other to create a chronic inflammatory, immunosuppressive, and tumor-promoting environment so that tumor cells can escape immune surveillance and survive against effector immune cells attack [47].

Under normal circumstances, autophagy degrades and recycles cytoplasmic components to maintain protein synthesis and other necessary metabolic functions, which is considered to be an endogenous defence mechanism [48–50]. However, autophagy can also promote or inhibit tumor progression in a variety of ways, depending on the background, which is thought to be a “double-edged sword” in tumors. Autophagy has been reported to regulate the interaction of tumor cells with various substances within surrounding milieu, especially immune system components that include B and T lymphocytes, dendritic cells, macrophages, and NK cells, as well as the cytokines and immunoglobulins they released [20]. Conversely, immune cells and the cytokines and antibodies they released could trigger autophagy dysfunction, which induces or suppresses tumorigenesis. Furthermore, the autophagy-mediated regulation of the immune system might strengthen or attenuate the effects of immunotherapy. Autophagy has been reported to enhance the effect of immunotherapy by exposing antigens to antigen-presenting cells (APCs) and CTL to initiate and execute the process of tumor recognition and elimination. Otherwise, a growing number of studies have demonstrated that autophagy could also attenuate the effects of immunotherapy by inducing an immunosuppressive atmosphere. This hinders the ability of effector immune cells to kill tumor cells [51]. These indicated that appropriate induction or inhibition of autophagy may represent a prospective therapeutic strategy when combined with chemotherapy, radiotherapy, and immunotherapy. But comprehensive and systematic analysis of the correlation between autophagy and tumor microenvironment has not yet been fully identified, which impedes the clinical development of autophagy-based activators or inhibitors.

In this study, we gathered ATGs to determine autophagy-related patterns through unsupervised consensus clustering. Five distinct autophagy-related patterns (ATPclusters) were identified with differential ATGs expression, survival benefit, TIME infiltration, and function annotation. In the TCGA-HNSCC cohort, ATPclusters A and B exhibited high TIME infiltration, while ATPclusters C–E were relatively less infiltrated with immune cells. Kaplan–Meier survival curves showed that the prognosis of patients with ATPclusters B and E was robustly better than that of patients with ATPclusters A, C, and D, which was not consistent with the findings related to TIME infiltration. We noticed that ATPclusterA was infiltrated with both effector immune cells and immunosuppressive cells, while ATPclusters B and E were highly infiltrated with activated CD8+ T cells and cytotoxic cells and less infiltrated with Treg, macrophages, and mast cells. Thus, we inferred that highly infiltrated immunosuppressive cells could counteract and impair effector immune cells to distinguish and eradicate abnormal tumor cells. As the opposite function of CTL and Treg in tumor immunity, a combined assessment of CTL and Treg infiltration has been comprehensively studied. The CTL/Treg ratio has finally been recognized as an independent prognostic factor in many tumor types [52, 53]. In our study, we found that CTL/Treg ratio was significantly higher in ATPclusters B and E than in ATPclusters A, C, and D, which well explains the mismatch of immune infiltration and survival analysis. Moreover, function annotation demonstrated that ATPcluster A was not only enriched in signaling pathways associated with inflammation but also induced in angiogenesis, EMT, and pan-fibroblast TGF-β response signaling pathways. This indicated that the stromal status was relatively activated in this pattern. The stromal status, which could reversibly shift between “loose” and “dense” status, is the key checkpoint for the appropriate localization and migration of T cells into the tumor parenchyma. As activated in stromal status, effector immune cells cannot effectively penetrate the stroma surrounding core tumor islets, leaving a large number of CTL strapped in the extracellular matrix, thus unable to perform their antitumor role of immune surveillance and eradication, allowing the tumor to progress in ATPcluster A, which was the characteristic of immune-excluded phenotype [54]. In addition to the lack of activated and priming T cells, ssGSEA of KEGG, HALLMARK, and specific signatures all revealed that immune tolerance and ignorance were fully induced in ATPclusters C and D, which was more likely to be to immune-desert phenotype [55]. Moreover, we also found that despite being highly infiltrated with effector immune cells, the antigen processing machinery, CD8+ T effector, and immune checkpoint signature, which were representative of immune activation, were also strikingly enriched in ATPclusters B and E, which was featured as immune-inflamed phenotype. Furthermore, the expression of immune activation, stromal activation, MHC molecules, and immune checkpoints showed similar distribution pattern as the function annotation. Recently, many teams have defined the non-inflamed or immune-suppressed tumors with immune-excluded and immune-desert phenotypes as “cold” tumors and tumors with immune-inflamed phenotype as “hot” tumors, which might be responsible for the clinical response to ICIs immunotherapy [56, 57]. Next, we systematically collected transcriptome datasets of HNSCC across GEO and ArrayExpress database and merged them as the meta-HNSCC cohort. We were amazed to find that all of the above results could be precisely validated in the meta-HNSCC cohort. All of these suggested that there are distinct autophagy-related patterns associated with signaling pathway enrichment, TIME infiltration, and immune phenotypes in HNSCC, which provides the possibility of combining autophagy activators or inhibitors with ICIs.

Accumulated evidence demonstrated that tumor patients with immune-inflamed phenotype will achieve durable responses and better overall survival when receiving ICIs treatment [55]. But, we also noticed that not all patients benefit from ICIs-targeting therapy, with an estimated response rate only modestly above the historical 10% response rate to traditional chemotherapies [37]. Immune tolerance to these tumors is still a major impediment in cancer immunotherapy. To improve the efficacy of immunotherapy and prolong survival after immunotherapy, we need to clarify the underlying mechanisms of immune tolerance. Many important factors affecting immune tolerance have been identified, such as hypo-infiltration of effector immune cells into the tumor parenchyma, imbalance between effector immune cells and immunosuppressive cells, abnormalities in the function and expression of MHC molecules, and lack of exposure to tumor antigens or epitopes leading to failure of antigen processing for T cells [58]. As highly correlated with TIME infiltration and immune phenotypes, the association between autophagy-related pattern and tumor mutation load was further investigated. The previous study demonstrated that the recognition of neo-antigens exposure, mainly triggered by somatic nonsynonymous mutations, was essential for the initiation of the antigen processing and activation of the adaptive tumor immunity cascade. Moreover, TMB, which could be easily assessed to replace the overall neo-antigen detection, has been identified as a potential biomarker for predicting the clinical outcome of ICIs treatment [59, 60]. In the TCGA-HNSCC cohort, we found that TMB was differentially distributed among five distinct autophagy-related patterns, with ATPcluster B showing the lowest TMB and ATPclusterD showing the highest TMB, which is not consistent with the results of TIME infiltration and function annotation. However, we were surprised to find that patients with high TMB in the TCGA-HNSCC cohort were associated with a worse prognosis than patients with low TMB. This indicated that TMB is harmful in the TCGA-HNSCC cohort, which contradicted our common sense. However, we then found that DNA damage repairing signaling pathways were significantly enriched in ATPclusters B and E, which might be another reason for the good prognosis in this pattern.

Then autophagy phenotype-related genes, which were extracted from the DEGs among distinct autophagy-related patterns, were subjected to LASSO-Cox regression analysis to establish a set scoring system to evaluate and quantify autophagy regulation pattern in individuals, which was referred to the autophagy phenotype-related signature (ATPscore). ATPscore was found to be differentially distributed among five distinct autophagy-related patterns. We further explored the effect of *SRPX*, one of the key genes of ATPscore in HNSCC cells and the results indicated the low expression of *SRPX* could significantly decrease the proliferation and migration of HNSCC.

In recent years, a great number of scoring systems have been established to predict the prognosis and clinical treatment response of HNSC patients. Yin et al. [61] developed a prognostic risk model for HNSCC based on m6AlncRNAs that could predict the prognosis and response to immunotherapy in HNSCC. Although they did a great work, their research included merely 400 samples and lacked external validation. Meanwhile, Wei et al. [62] identified genes associated with ferroptosis prognostic score by weighted correlation network analysis (WGCNA) and LASSO, and further constructed a clinical prognostic model of ferroptosis-related prognostic risk score (FPRS). However, their research did not pay much attention to the function of score-related genes. In our research, we included 6 datasets as well as over 1000 research samples and we also verified the function of *SRPX* in various cell lines, which make our conclusions more credible.

Moreover, in the TCGA-HNSCC, microarray-HNSCC, and IMvigor210 (mUC) cohorts, we were pleased to find that the low ATPscore group was significantly enriched in immune activation relevant signaling pathways and deactivated in stromal relevant signaling pathways, which is characteristic of a “hot” tumor. Thus, the opposite phenomenon was seen in the high ATPscore group, corresponding to “cold” tumor. In addition, we validated these in the IMvigor210 (mUC) cohort to find that immune-inflamed phenotype had the lowest ATPscore, while immune-desert and immune-excluded phenotypes had the higher ATPscore, which indicated the successful model construction. All the above results revealed that ATPscore was not only a reliable tool to assess autophagy-related pattern but also was potent to effectively evaluate TIME infiltration and immune phenotype in individuals. In addition, we found that patients with good clinical outcomes, as well as low malignancy clinicopathological traits and molecular subtypes were more likely to be in the low ATPscore group, while the opposite patterns were observed in the high ATPscore group. In the IMvigor210 (mUC) cohort, we found that patients with GU and TCGA II molecular subtypes, as well as IC2 phenotypes, which was reported to highly respond to ICIs-targeting immunotherapy, were robustly concentrated in the low ATPscore group, and rarely observed in the high ATPscore group [27, 37]. Moreover, we found that TMB did not differ between the high and low ATPscore groups in the TCGA-HNSCC cohort. But the patients in the high ATPscore group were more likely to have a *TP53* mutation, again indicating the role of ATPscore in predicting the ICIs immunotherapy response, as mutation status of *TP53* could have predictive value for immunotherapy in patients with HNSCC [63]. Although TMB did not perform well in the TCGA-HNSCC cohort, we found here in the IMvigor210 (mUC) cohort that ATPscore was negatively correlated with TMB and patients with the low ATPscore group were more likely to be patients with high TMB, which was consistent with findings in GU molecular subtype with high mutation load. Moreover, Kaplan–Meier survival curves showed that the combination of ATPscore and TMB could significantly improve the predictive value when compared with TMB or ATPscore alone, with patients with low ATPscore and high TMB having the best prognosis. Finally, we found that patients with low ATPscore were more likely to benefit from ICIs treatment and ICIs targeting immunotherapy responders had a lower ATPscore. According to all these findings, autophagy-related patterns and ATPscore were found to be significantly correlated with three main factors: pre-existing activated CTL or immunoreactivity, activation of the EMT/TGF-β signaling pathway or stromal status, and tumor neo-antigen or TMB levels to influence the clinical outcome of ICIs immunotherapy.

## Conclusion

We comprehensively and systematically assessed distinct autophagy-related patterns and established a set scoring system ATPscore that could represent them and was associated with TIME infiltration, immune phenotypes, molecular subtypes, genetic variations, clinical outcome of ICIs targeting immunotherapy, etc. The effect of *SRPX* was also verified in the HNSCC cell line. More importantly, this study has yielded novel insights into the combination of autophagy-based inducers or inhibitors with various therapeutic strategies such as immunotherapy for clinical application in HNSCC.

## Supplemental Data

Supplementary data are available at the following link: https://www.bjbms.org/ojs/index.php/bjbms/article/view/9094/2801.
